# SPEN, a new player in primary cilia formation and cell migration in breast cancer

**DOI:** 10.1186/s13058-017-0897-3

**Published:** 2017-09-06

**Authors:** Stéphanie Légaré, Catherine Chabot, Mark Basik

**Affiliations:** 1 Division of Experimental Medicine, Department of Oncology and Surgery, McGill University, Montréal, PQ H3A 0G4 Canada; 2 Segal Cancer Center, Lady Davis Institute for Medical Research, Sir Mortimer B. Davis Jewish General Hospital, McGill University, Montréal, PQ H3T 1E2 Canada; 3 Department of Surgery and Oncology, Lady Davis Institute for Medical Research, 3755 Cote Ste Catherine, Montréal, QC H3T 1E2 Canada

**Keywords:** Primary cilia, Cell migration, Breast cancer, Metastasis

## Abstract

**Background:**

The primary cilium is a microtubule-based and nonmotile organelle functioning as a cellular antenna that is involved in the regulation of cell proliferation, differentiation, and migration. In breast cancer cells, the primary cilium is a structure that decreases in incidence with increasing degrees of transformation and may be biologically more important in estrogen receptor (ERα)-negative breast cancer cells. Split ends (SPEN) is an ERα corepressor that we have identified as a tumor suppressor protein in ERα-positive breast cancer cells whose hormone-independent roles in breast cancer have never been explored.

**Methods:**

We determined the hormone-independent transcriptional program regulated by the ERα cofactor SPEN in breast cancer using DNA microarrays. The biological functions regulated by SPEN independently of hormones were studied in vitro in ERα-positive and ERα-negative breast cancer cells. Finally, we examined the clinical relevance of SPEN expression in cohorts of breast cancer samples with outcome data.

**Results:**

We found that *SPEN* is coexpressed with a number of genes involved in ciliary biology, including the ciliogenic transcription factor RFX3, in a hormone-independent manner. SPEN reexpression in T47D cells containing a nonsense mutation in *SPEN* restored the primary cilium, whereas its knockdown in MCF10A and Hs578T cells considerably decreased primary cilia levels. We also report that SPEN regulates migration in breast cells, but only in those harboring primary cilia, and that KIF3A silencing, a critical factor in primary cilia, partially reverses SPEN’s effects, suggesting that SPEN may coordinate cellular movement through primary cilia-dependent mechanisms. Finally, we found that high *SPEN* RNA levels were predictive of early metastasis in two independent cohorts of 77 (HR 2.25, *P* = 0.03) and 170 (HR = 2.23, *P* = 0.004) patients with ERα-negative breast cancer.

**Conclusions:**

Together, our data demonstrate a role for SPEN in the regulation of primary cilia formation and cell migration in breast cancer cells, which may collectively explain why its expression is associated with time to metastasis in cohorts of patients with ERα-negative breast cancers.

**Electronic supplementary material:**

The online version of this article (doi:10.1186/s13058-017-0897-3) contains supplementary material, which is available to authorized users.

## Background

Primary cilia are microtubule-based, nonmotile cell appendages protruding from most mammalian cells that were identified more than 100 years ago as vestigial organelles [1]. Primary cilia extend from the mother centriole into the extracellular environment and function as antennas sensing and transducing signals from the external milieu into cells [2–5]. Signaling through primary cilia regulates a number of biological processes, including cell polarity, migration, and differentiation, in part through the activation of primary cilia-associated components, including members of the Hedgehog, mTORC1, platelet-derived growth factor receptor (PDGFR), and Wnt signaling pathways [2, 5]. Primary cilia also play important roles during development, with defects in the establishment of primary cilia due to mutation- or transcription-driven alterations in ciliary genes being responsible for a number of ciliopathies and disorders, such as polycystic kidney disease, situs inversus, and Bardet-Biedl syndrome. In cancer tissues, including pancreatic, prostate, and breast cancers, primary cilia are decreased in incidence compared with normal-matched tissues [6–9]. Recently, a study conducted with a collection of breast cancer cell lines and tissues revealed that primary cilia are lost at a very early stage during breast cancer development, raising the possibility that they may have tumor-suppressive functions in breast cancer and possibly other cancer types [10, 11]. Yet, in another study, researchers reported that the presence of primary cilia in cancer cells correlates with poor prognosis and lymph node metastasis in patients with pancreatic cancer [12]. Indeed, primary cilia are still poorly characterized and studied organelles whose functions as sensing and signaling platforms in normal and transformed mammalian cells are only beginning to be explored.

Split ends (SPEN), also known as SMRT/HDAC1-associated repressor protein, is a large nuclear protein with essential roles in transcriptional regulation and chromosome X inactivation [13–16]. SPEN contains four N-terminal RNA recognition motifs and a highly conserved Spen paralogue and orthologue C-terminal domain (SPOC) that is required for its transcriptional repression of nuclear hormone receptors (HRs) [17]. Given the presumptive role of SPEN in endocrine response, our laboratory has characterized SPEN functions in estrogen receptor alpha (ERα)-positive breast cancers and established SPEN as a transcriptional corepressor of the ERα, tumor suppressor gene, and candidate predictive biomarker of tamoxifen response in hormone-dependent breast cancers [13].

In the present study, we examined the hormone-independent transcriptional program and functions regulated by SPEN in breast cancer and found that *SPEN* is significantly coexpressed with genes involved in ciliary biology. We demonstrate that SPEN positively regulates primary cilia formation and cell migration in breast cancer, possibly via the transcriptional regulation of *RFX3*, a ciliogenic transcription factor [18–21]. Interestingly, we found that *SPEN* knockdown attenuates cell migration in breast cancer cells when accompanied with a concomitant decrease in primary cilia levels, indicating that SPEN may regulate cellular movement through primary cilia-dependent mechanisms. We also report that high *SPEN* expression levels are predictive of early metastasis in patients with ERα-negative but not ERα-positive breast cancers. Altogether, our results establish SPEN as a new player in primary cilia formation and cell migration in breast cells, two functions that may collectively explain why *SPEN* expression is associated with time to metastasis in patients with ERα-negative breast cancers.

## Methods

### Cell lines

All cell lines were obtained from the American Type Culture Collection (ATCC; Manassas, VA, USA). T47D clones (CTL and SPEN) were stably transfected with control and *SPEN*-expressing vectors as previously described and were cultured in Gibco DMEM (Life Technologies, Carlsbad, CA, USA) supplemented with 10% FBS (Wisent Bio Products, Saint-Bruno, PQ, Canada) and G418 (500 μg/ml) under conditions specified by the manufacturer. BT20 and MDA-MB-436 cells were cultured in Eagle’s minimum essential medium and RPMI 1640 (ATCC), respectively, supplemented with 10% FBS. MCF10A cells were maintained in RPMI 1640 (ATCC) containing 10% FBS, 10 μg/ml insulin, 0.5 μg/ml hydrocortisone, and 20 ng/ml human epidermal growth factor. MCF10A-CT cells were maintained in Gibco DMEM/Ham’s F-12 Nutrient Mixture (Life Technologies) containing 5% horse serum, 10 μg/ml insulin, 0.5 μg/ml hydrocortisone, 20 ng/ml human epidermal growth factor, and 100 ng/ml cholera toxin (CT).

### DNA microarray expression profiling and analysis

Expression profiling was conducted according to the manufacturer’s instructions and following the One-Color Microarray-Based Gene Expression Analysis protocol (Agilent Technologies, Santa Clara, CA, USA). Briefly, the integrity and concentration of the input RNA was evaluated with the Agilent 2011 Bioanalyzer (Agilent Technologies). Total RNA (100 ng) was reverse-transcribed into complementary RNA (cRNA), amplified, and labeled with cyanine 3 dye. The resulting labeled cRNA was purified using the RNeasy Mini Kit (QIAGEN Sciences, Germantown, MD, USA) according to the instructions of the manufacturer and hybridized to a Sure Print G3 Human Gene Expression 8 × 60 K microarray (Agilent Technologies) for 17 h at 65 °C. The array was then washed and scanned on the Agilent DNA microarray scanner at a resolution of 3 μm. Images were extracted and normalized with Feature Extraction version 9.5 software (Agilent Technologies). Expression values of three biological RNA replicates for each probe in the expression array were analyzed using GeneSpring GX software (Agilent Technologies). The microarray data from this study are available on ArrayExpress under accession numbers [E-MTAB-4974 and E-MTAB-4875].

### Statistical analysis

Microarray expression data were processed with GeneSpring GX software. Data were normalized to the 75th percentile of all values on the microarrays and to the median expression levels of all samples. The normalized gene expression data were filtered on flags, and all detected and undetected flags were allowed to pass the filter and were included in the analysis. The expression profiles of genes differentially expressed by more than 1.5-fold on the basis of three biological replicates were compared using unpaired *t* tests.

### Ingenuity Pathway Analysis

Microarray analyses were performed using the Ingenuity Pathway Analysis software (QIAGEN Bioinformatics, Redwood City, CA, USA). Genes upregulated by 2.00-fold (*P* < 0.05) in T47D-SPEN compared with its control (T47D-CTL) and downregulated by 2.00-fold (*P* < 0.05) in SPEN-silenced compared with control MCF10A cells were considered for further analyses.

### Immunoprecipitation

Cells were rinsed in PBS, harvested, and lysed in buffer (250 mM NaCl, 0.5% Nonidet P-40, 5 mM ethylenediaminetetraacetic acid [EDTA], 50 mM Tris) freshly supplemented with protease inhibitors (sodium fluoride, sodium orthovanadate, phenylmethylsulfonyl fluoride, aprotinin, and leupeptin). After micro-bicinchoninic acid quantification, 0.5 mg of proteins was incubated overnight with 1 μg of anti-SPEN antibody (HPA015825; Sigma-Aldrich, St. Louis, MO, USA), RFX3 (TA505916; OriGene Technologies, Rockville, MD, USA), or immunoglobulin G (IgG) control antibody (ab46540-1; Abcam, Cambridge, MA, USA). Protein lysates were incubated for 3 h with protein A beads (45000116; GE Healthcare Bio-Sciences, Piscataway, NJ, USA), centrifuged (4000 rpm), and washed three times with 400 μl of lysis buffer. Precipitated beads were then incubated at 95 °C for 7 minutes in 60 μl of the loading buffer, followed by centrifugation at 13,000 rpm for 1 minute. SPEN-knockdown post-small interfering RNA (post-siRNA) transfection was confirmed twice for each of the four cell lines under study. Immunoprecipitation assays for the measurement of RFX3 protein expression levels were performed at least three times.

### siRNA transfections

All siRNA transfections were done using Lipofectamine RNAiMAX Transfection Reagent (Life Technologies) following the manufacturer’s protocol. Briefly, 2.5 × 10^5^ cells were plated in six-well plates, and the transfection complex (containing 9 μl of Lipofectamine RNAiMAX Transfection Reagent and 30 pmol of siRNAs) was added to cells 24 h later. After another 72 h, transfected cells were analyzed for RNA and/or protein expression by qRT-PCR and Western blotting, respectively. A fraction of transfected cells was also analyzed by fluorescence-activated cell sorting for cell migration, or it was plated in low serum-containing medium for primary cilia quantification by immunofluorescence. All experiments were done within 6 days posttransfection.

### Immunofluorescence

For immunofluorescence staining of cultured cells, cells were seeded onto coverslips in six-well plates (T47D cells) or into eight-chamber cell culture slides (siRNA-treated cells) and allowed to grow for 3 days. T47D cells were cultured in hormone-depleted medium, whereas all other cell lines were grown in their respective media supplemented with 1% FBS. Cells were washed with PBS, then fixed in 4.0% paraformaldehyde in DMEM with 10% FBS for 10 minutes, and then incubated in blocking solution (1% goat serum, 0.1% Triton X-100 in PBS) for 30 minutes. Cells were then incubated in primary antibodies for 1 h at room temperature. Primary antibodies used were mouse antiacetylated α-tubulin (1:4000 dilution, clone 6-11B-1; Sigma-Aldrich) and rabbit γ-tubulin (1:1000 dilution; Sigma-Aldrich). After being washed four times for 10 minutes each with PBS, cells were incubated with secondary antibodies (donkey antimouse Alexa Fluor 594 and goat antirabbit Alexa 488; Invitrogen/Life Technologies) for 1 h at room temperature. Cells were incubated for 4 minutes with 4′,6-diaminodino-2-phenylindole (5 μg/ml; Sigma-Aldrich) and washed four times with PBS for 10 minutes. Slides were mounted with CureMount II mounting medium (Leica Biosystems, Buffalo Grove, IL, USA). Images were obtained with a × 63 or × 100 lens objective. Primary cilia were identified as such when a ciliary acetylated α-tubulin structure, a marker of the axoneme, was colocalized to γ-tubulin staining, a marker for centrosomes. No minimal length cutoff was used. Primary cilia were manually counted in at least 15 independent microscopic fields (except for the KIF3A-knockdown assay, in which 10 independent microscopic fields were examined) per slide, and the frequency of primary cilia was calculated by dividing the number of cilia by the number of counted nuclei. Primary cilia levels were quantified in at least three independent experiments for each cell line (>750 cells were counted in total per experimental condition, except for lines of fibroblasts, in which approximately 400 cells were counted because of slow growth rates).

### Western blotting

Subconfluent cells were collected by trypsinization, washed in ice-cold PBS, and lysed in lysis buffer freshly supplemented with protease inhibitors. Lysates were then centrifuged for 15 minutes at 4 °C. Supernatants were subjected to Bradford quantification, and 50 μg of proteins were loaded and run by SDS-PAGE in a 10% gel for 1 h. Proteins were transferred to nitrocellulose membranes and incubated with antibodies for RFX3 (TA505916; OriGene Technologies), KIF3A (D7G3, #8507; Cell Signaling Technology, Danvers, MA, USA), sperm flagellar 2 (SPEF2, catalog number ab57761; Abcam), and α-tubulin or β-actin. All Western blot experiments were performed at least three times.

### Fluorescence activated cell sorting

For cell cycle analyses, cells were detached with trypsin, washed in PBS supplemented with 5 mmol/L EDTA, suspended in a fixing solution (1 ml of PBS, 5 mmol/L EDTA for 3 ml of 100% ethanol), and incubated at −20 °C for at least 24 h. Then cells were washed with PBS/EDTA and resuspended in 1 ml of staining solution (PBS, 50 mg/ml propidium iodide, and 20 mg/ml RNase A). The analysis was performed using BD CellQuest (BD Biosciences, San Jose, CA, USA), ModFit (Verity Software House, Topsham, ME, USA), and FlowJo (FlowJo, Ashland, OR, USA) software. At least three independent experiments were performed for each assay.

### Intersection probabilities

To determine the statistical significance of intersection between two lists of genes, we assessed the probability of this intersection to occur by performing 10,000 independent simulations with randomly selected lists of genes of the same size. *P* values were calculated using a hypergeometric test.

### Migration assays

Cell migration was assessed using a Boyden chamber assay. For these experiments, 5 × 10^5^ cells (T47D cells) or 2.5 × 10^5^ cells (all other cell lines) were seeded onto the upper well of a Costar Transwell chamber (8 μM; Corning Life Sciences, Tewksbury, MA, USA) in serum-free medium, except for T47D cells. The latter were seeded in complete medium, which was replaced with 0% FBS-containing medium 24 h later. For all cell lines except T47D clones, cells that had migrated to the bottom side of the membrane were fixed in 70% ethanol and stained with crystal violet 24 h after plating. This was done 72 h after seeding for T47D cells. After being stained, nonmigrating cells in the upper chamber were removed using a cotton-tipped applicator. The membranes were mounted onto object slides, and six random fields per slide were counted with a × 10 or × 20 lens objective. At least three independent experiments were performed for each assay.

## Results

### SPEN is coexpressed with genes involved in cilium biology

**Fig. 1:**
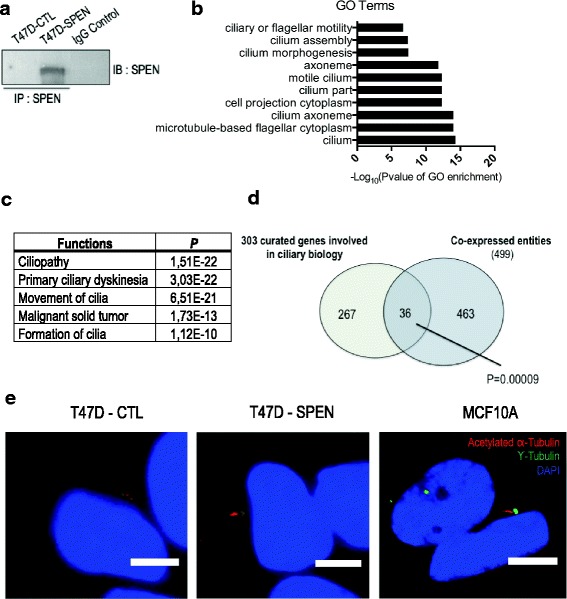
*SPEN* is coexpressed with genes involved in ciliary biology. **a** Immunoprecipitated (IP) SPEN protein levels in T47D-CTL and T47D-Spen clones. Immunoprecipitation with nonspecific rabbit IgG was done as a negative control. **b** Top ten GO terms from the analysis performed with the list of genes associated with SPEN reexpression in T47D-SPEN cells in three different conditions, classified from the least to the most significant. **c** Top significantly enriched functions from the Ingenuity Pathway Analysis of the list of genes coexpressed with *SPEN*. **d** Venn diagram showing the intersection between the list of genes coexpressed with *SPEN* in T47D clones and a curated list of genes involved in ciliary biology. **e** Representative images of primary cilia in T47D-SPEN and MCF10A cells (positive control). Primary cilia were identified by coimmunofluorescence for acetylated α-tubulin (*red*) and γ-tubulin (*green*), markers of the ciliary axoneme and centrosome, respectively (scale bar = 5 μm). The microarray data for this study can be found on ArrayExpress under the accession number [E-MTAB-4974]. *DAPI* 4′,6-Diaminodino-2-phenylindole, *GO* Gene Ontology, *IgG* Immunoglobulin G, *SPEN* Split ends

We have previously established *SPEN* as a tumor suppressor gene and candidate predictive biomarker of tamoxifen response in ERα-positive breast tumors [13]. Our work demonstrated that SPEN overexpression in T47D cells attenuates hormonal responses and potentiates the antiproliferative and proapoptotic effects of the antiestrogen tamoxifen. In this recently published study [13], we largely explored SPEN functions in ERα-positive breast cancer cells under estrogenic conditions. To determine the hormone-independent transcriptional program regulated by SPEN, we performed gene expression analyses on a previously described SPEN-overexpressing clone (T47D-SPEN) and that of its empty vector-transfected control (T47D-CTL) grown in three different conditions: a hormone-free condition and in the presence of an ERα agonist (estradiol) or an ERα antagonist (tamoxifen). Genes upregulated by at least 2.0-fold (*P* ≥ 0.05) in T47D-SPEN relative to T47D-CTL in all experimental conditions and strongly coexpressed (*R* ≥ 0.942 *P* = 0.005) with *SPEN*, regardless of the treatment, were considered as regulated by SPEN, independently of hormones. A Gene Ontology analysis performed with the list of 499 genes coexpressed with *SPEN* revealed marked enrichment for terms associated with the cilium (*P* = 1.64E-19), cilium axoneme (*P* = 1.05E-18), and cilium part (*P* = 6.85E-17) (Fig. 1a and b). Consistently, the biological functions “ciliopathy” (*P* = 1.51E-22), “primary ciliary dyskinesia” (*P* = 3.03E-22), and “movement of cilia” (*P* = 6.51E-21) were significantly enriched in Ingenuity Pathway Analysis, suggesting that SPEN may regulate the expression of a number of genes relevant to the cilium (Fig. 1c). We also cross-referenced our microarray data with a publicly available and curated list of 303 genes involved in cilium biology and found an overlap greater than that predicted by chance (*P* = 0.00009), implying that a significant number of genes functionally relevant to primary cilia may be regulated by SPEN (Fig. 1d and Additional file 1: Table S1) [22].

### SPEN regulates primary ciliogenesis in breast cells

The marked enrichment for cilium-related genes observed in our microarray analysis led us to investigate whether SPEN reexpression is accompanied by an increased prevalence of primary cilia in T47D cells. A study conducted by Yuan et al. [9] on a panel of breast cancer cell lines and tissues showed that primary cilia are undetectable in luminal breast cancer cells, including T47D and MCF-7 cells, whereas they are highly prevalent in the nontumorigenic MCF10A cells and breast cancer cell lines of the basal subtype that typically possesses mesenchymal characteristics. To evaluate whether SPEN overexpression restores the primary cilium in T47D cells, we assessed primary cilia incidence in T47D-SPEN and T47D-CTL clones by immunofluorescence, using antibodies for acetylated α-tubulin to visualize cilia and γ-tubulin to identify associated centrosomes, both of which are required to be adjacent/juxtaposed for the identification of primary cilia [23]. MCF10A cells, which are known to harbor high levels of primary cilia, were used as a positive control. As anticipated, no primary cilia was detected in T47D-CTL. Very interestingly, SPEN reexpression restored the primary cilium in 1.80% of T47D cells, a value that is within the range reported for normal luminal breast cells and breast cancer cells (Fig. 1e) [9, 10].

**Fig. 2:**
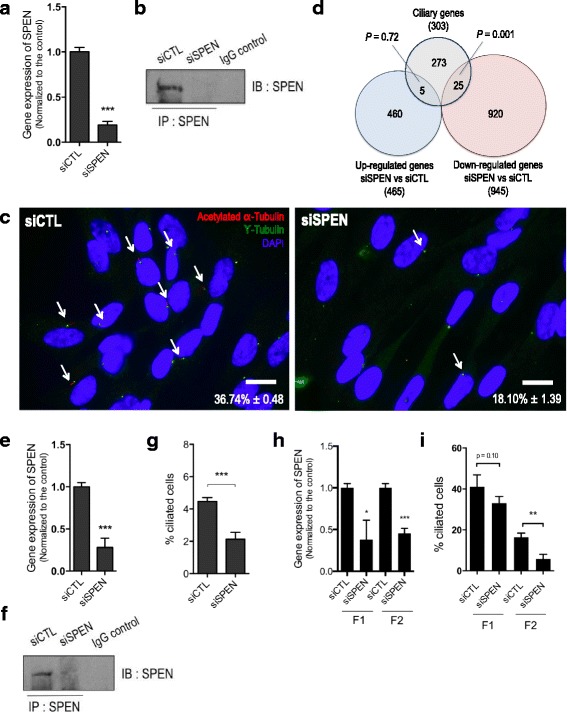
SPEN regulates primary cilia formation. **a** qRT-PCR of SPEN expression in MCF10A treated with siRNA control or siRNA for *SPEN*. Represented is the mean (±SEM) expression value of *SPEN* in at least three biological replicates, normalized to siRNA control-treated cells. **b** Immunoprecipitated (IP) SPEN protein levels in MCF10A cells treated with siRNA control or siRNA against *SPEN*. Immunoprecipitation with nonspecific rabbit IgG was done as a negative control. **c** Representative images of primary cilia in MCF10A cells treated with siRNA control or siRNA against *SPEN*. *Arrows* point to primary cilia (scale bar = 5 μm). **d** Venn diagram showing the intersection of the list of genes up- and downregulated by SPEN knockdown in MCF10A cells and a curated list of genes involved in ciliary biology. The microarray data for this study can be found on ArrayExpress under the accession number [E-MTAB-4975]. **e** qRT-PCR of *SPEN* expression in Hs578T cells treated with siRNA control or siRNA for *SPEN*. Represented is the mean (±SEM) expression value of *SPEN* in at least three biological replicates, normalized to siRNA control-treated cells. **f** Immunoprecipitated SPEN protein levels in Hs578T cells treated with siRNA control or siRNA against *SPEN*. Immunoprecipitation with nonspecific rabbit IgG was done as a negative control. **g** Percentage of ciliated cells in Hs578T cells treated with siRNA control or siRNA targeting *SPEN*. **h** Fraction of ciliated cells in two lines of fibroblasts treated with siRNA for *SPEN*, normalized to siRNA control-treated cells. **i** qRT-PCR of *SPEN* expression in two lines of fibroblast-treated siRNA for *SPEN*, normalized to siRNA control-treated cells. Represented is the mean (±SEM) expression value of *SPEN* in three biological replicates. *** *P* ≤ 0.005; ** *P* ≤ 0.01; * *P* ≤ 0.05). *DAPI* 4′,6-Diaminodino-2-phenylindole, *IgG* Immunoglobulin G, *siRNA* Small interfering RNA, *SPEN* Split ends

To confirm the role of SPEN in the modulation of ciliogenesis, we then silenced SPEN expression in the nontumorigenic MCF10A cells, which show high endogenous levels of SPEN as well as high prevalence of primary cilia. Importantly, these experiments were performed with MCF10A cells maintained in the absence of CT, a factor that elevates cAMP levels and mitogen-activated protein kinase signaling and that is normally required for the growth of MCF10A cells [24]. We observed that MCF10A cells grown in the absence of CT adopt an elongated, mesenchyme-like phenotype, which contrasts with the epithelial morphology of MCF10A cells maintained in the presence of CT (Additional file 2: Figure S1a and b). Even further, we found that addition of CT almost completely abolished primary cilium formation in MCF10A cells (1.46 ± 0.41% vs 36.70 ± 0.41% for cells grown in the absence of CT) (Additional file 2: Figure S1c and d), possibly because it strongly stimulates proliferation and cell cycle entry, two conditions known to be incompatible with the formation of primary cilia (Additional file 2: Figure S1e and f) [25]. For this reason, we used MCF10A cells grown in the absence of CT to investigate further SPEN functions in primary cilia formation. Gene silencing by siRNA against *SPEN*  was validated at the RNA and protein levels (Fig. 2a and b). We found that the RNA interference (RNAi)-mediated silencing of *SPEN* causes a substantial and significant decrease in primary cilia incidence in MCF10A cells (Fig. 2c). Interestingly, transcriptional profiling analyses conducted on MCF10-A-siRNA-SPEN cells revealed that *SPEN* silencing modulates the expression of a number of ciliary genes. Indeed, we noted a significant enrichment for genes relevant to ciliary biology among the list of genes downregulated (*P* = 0.001) but not upregulated (*P* = 0.72) subsequent to *SPEN* knockdown, further supporting a role for SPEN in the transcriptional control of cilium-related genes (Fig. 2d and Additional file 1: Table S1).

Interestingly, Ingenuity Pathway Analysis revealed that SPEN reexpression in T47D cells is accompanied by increased activation of Sonic hedgehog (SHH) (Z-score 2.62, *P* = 1.06E-04) and GLI1 (Z-score 2.39, *P* = 3.46E-03), two components of the Hedgehog pathway as well as increased signaling downstream of Pdgf (complex) (Z-score 1.20, *P* = 3.04E-02) compared with T47D-CTL. A similar analysis performed with the list of genes expressed with *SPEN* in siRNA-treated MCF10A cells predicted that *SPEN* knockdown would attenuate activation of the SHH (Z-score −2.36, *P* = 2.02E-05) and Pdgf (complex) (Z-score −1.97, *P* = 2.48E-03) components. Taken together, these data demonstrate that SPEN regulation of primary cilia formation is consistently accompanied by the modulation of transduction pathways that are signaling through the primary cilium, including the Hedgehog and PDGFR pathways.

We also found that *SPEN* knockdown in the basal-like breast cancer cell line Hs578T considerably decreased primary cilia levels (Fig. 2e–g). Moreover, *SPEN* knockdown in two independent lines of fibroblasts also diminished primary cilia abundance (Fig. 2h and i). Taken together, these results suggest that SPEN may regulate primary ciliogenesis in cancer cell lines from the breast and possibly in several additional cell types expressing primary cilia.

### SPEN regulates migration in breast cells

**Fig. 3:**
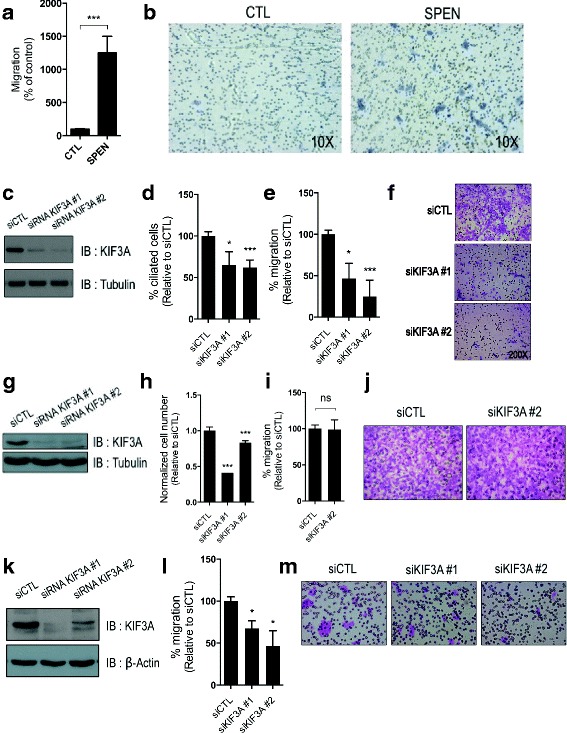
SPEN regulates migration in T47D cells. **a** and **b** Cell migration analysis of T47D-CTL and T47D-SPEN as evaluated by Boyden chamber assay. Represented is the mean (±SEM) percentage migration, normalized to the control, in least three biological replicates. Representative pictures showing cell migration in T47D-CTL and T47D-SPEN cells as evaluated by Boyden chamber assay. **c** Representative immunoblot (IB) showing KIF3A protein levels in MCF10A cells treated with siRNA control or siRNA against *KIF3A*. **d** Percentage of ciliated cells in MCF10A cells treated with siRNA control or siRNA targeting *KIF3A*. **e** and **f** Cell migration analysis of MCF10A cells treated with siRNA control or siRNA targeting *KIF3A* as evaluated by Boyden chamber assay. Represented is the mean (±SEM) percentage migration, normalized to the control, in least three independent experiments. Representative pictures showing cell migration in MCF10A cells treated with siRNA control or siRNAs for *KIF3A* as evaluated by Boyden chamber assay. **g** Representative IB showing KIF3A protein levels in MDA-MB-436 cells treated with siRNA control or siRNA against *KIF3A*. **h** Relative cell number of MDA-MB-436 cells treated with siRNA control or siRNA targeting *KIF3A* 72 h after transfection. **i** and **j** Cell migration analysis of MDA-MB-436 cells treated with siRNA control or siRNA targeting *KIF3A* as evaluated by Boyden chamber assay. Represented is the mean (±SEM) percentage migration, normalized to the control, in least three independent experiments. Representative pictures showing cell migration in MDA-MB-436 cells treated with siRNA control or siRNA for KIF3A as evaluated by Boyden chamber assay. **k** Representative IB showing KIF3A protein levels in T47D-SPEN cells treated with siRNA control or siRNA against *KIF3A*. **l** and **m** Cell migration analysis of T47D-SPEN cells treated with siRNA control or siRNA targeting *KIF3A* as evaluated by Boyden chamber assay. Represented is the mean (±SEM) percentage migration, normalized to the control, in least three independent experiments. Representative pictures showing cell migration in T47D-SPEN cells treated with siRNA control or siRNA for *KIF3A* as evaluated by Boyden chamber assay. *siRNA* Small interfering RNA, *SPEN* Split ends

Often referred to as *cellular GPS*, primary cilia are structures extending from the cell surface that have important roles in cell positioning and movement [26–28]. Indeed, several groups have reported that primary cilia are oriented in parallel to substrates and aligned in the direction of cell movement in wound-healing assays [3, 28]. Given the established role of primary cilia in cellular migration, we then investigated whether the modulation of SPEN expression in breast cells affects cell movement. We found that SPEN reexpression in T47D cells significantly increases cell migration in Boyden chamber assays (Fig. 3a and b). To test whether SPEN effects on migration may be mediated by primary cilia, we then disrupted cilia formation through the RNAi-mediated silencing of *KIF3A*, which encodes for a structural protein essential to ciliogenesis [29]. We first showed that *KIF3A* knockdown causes a significant decrease in primary cilia levels in the ciliated MCF10A cells, together with a decrease in cell migration (Fig. 3b–f). In addition, we report that the RNAi-mediated silencing of *KIF3A* in MDA-MB-436 cells does not affect cell migration, although only one siRNA was used, owing to unspecific effects interfering with cell survival and adhesion (Fig. 3g–j). Of note, silencing *KIF3A* expression in T47D-SPEN cells reversed the effect of SPEN on migration, suggesting that SPEN may affect cellular movement through its regulation of primary cilia levels in T47D cells (Fig. 3k–m). Moreover, RNAi-mediated silencing of *SPEF2*, which encodes for a flagellar protein required for ciliary function whose protein and RNA expression are also increased in T47D-SPEN cells compared with T47D-CTL cells, also resulted in marked inhibition of cellular migration in T47-SPEN (Additional file 3: Figure S2a–c) [22].

**Fig. 4:**
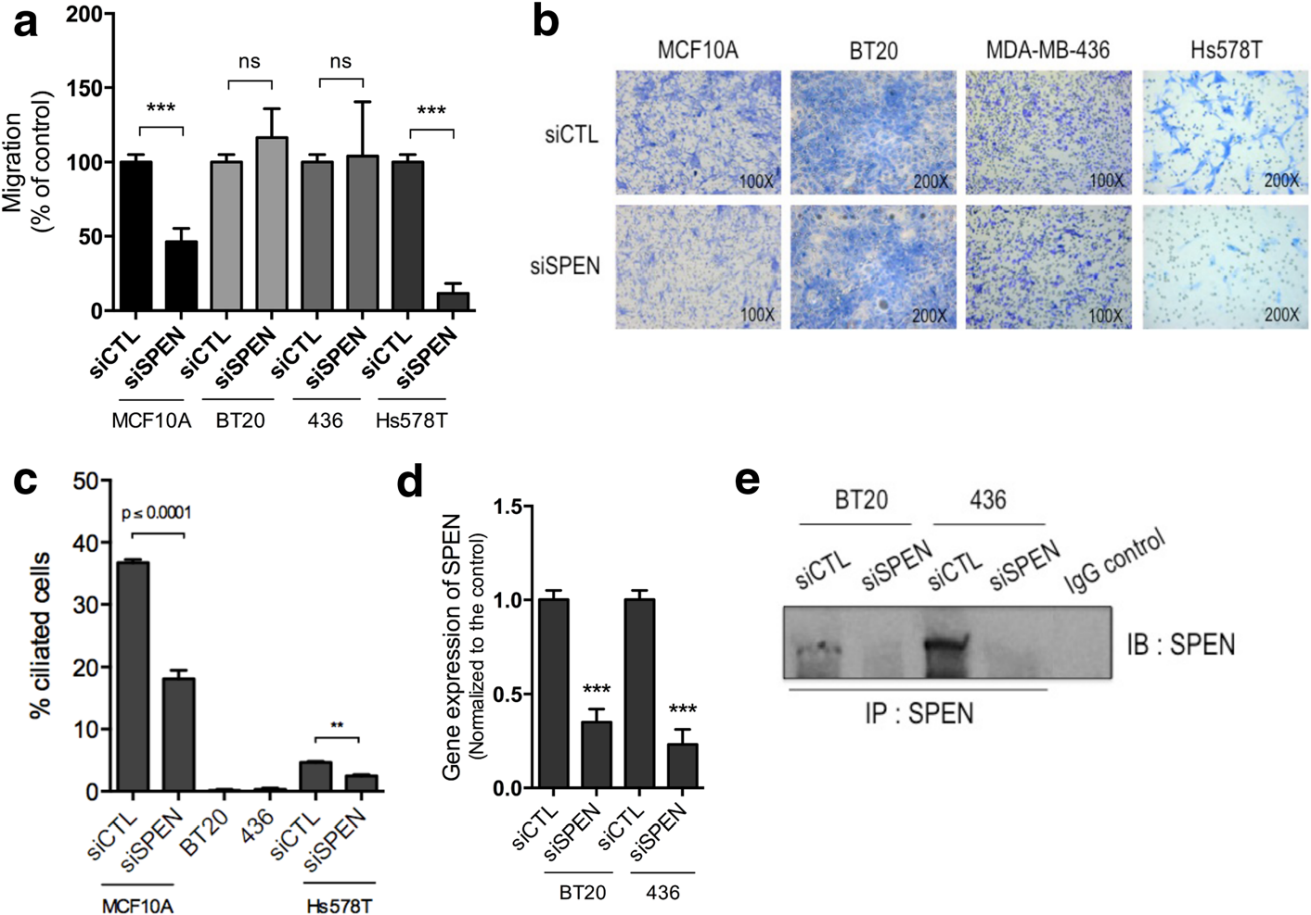
SPEN regulates migration in ciliated breast epithelial cells. **a** and **b** Cell migration analysis of MCF10A, BT20, MDA-MB-436, and Hs578T cells treated with siRNA control or siRNA targeting *SPEN* as evaluated by Boyden chamber assay. Represented is the mean (±SEM) percentage migration, normalized to the control, in least three independent experiments. Representative pictures showing cell migration in MCF10A, BT20, MDA-MB-436, and Hs578T cells treated with siRNA control or siRNA for *SPEN* as evaluated by Boyden chamber assay. **c** Percentage of ciliated cells in several lines of breast epithelial cells. **d** qRT-PCR of *SPEN* expression in BT20 and MDA-MB-436 (436) cells treated with siRNA for *SPEN*, normalized to siRNA control-treated cells. Represented is the mean (±SEM) expression value of *SPEN* in three biological replicates. **e** Immunoprecipitated (IP) SPEN protein levels in BT20 and MDA-MB-436 (436) cells treated with siRNA control or siRNA against *SPEN*. Immunoprecipitation with nonspecific rabbit IgG was done as a negative control. *** *P* ≤ 0.005; ** *P* ≤ 0.01; * *P* ≤ 0.05. *IB* Immunoblot, *IgG* Immunoglobulin G, *siRNA* Small interfering RNA, *SPEN* Split ends

To strengthen the link between SPEN, primary cilia, and cell migration, we then assessed whether *SPEN* knockdown affects cellular movement in the ciliated MCF10A and Hs578T cells as well as in the nonciliated BT20 and MDA-MB-436 breast cancer cell lines. We found that *SPEN* silencing considerably decreased cell migration in MCF10A and Hs578T cells but not in BT20 and MDA-MB-436 cells, supporting the role of SPEN in the regulation of cell migration through primary cilia-dependent mechanisms (Fig. 4a–e). Together, these findings suggest that SPEN possibly affects cellular movement through its regulation of primary cilia formation.

### SPEN is associated with metastasis in breast cancer

**Fig. 5:**
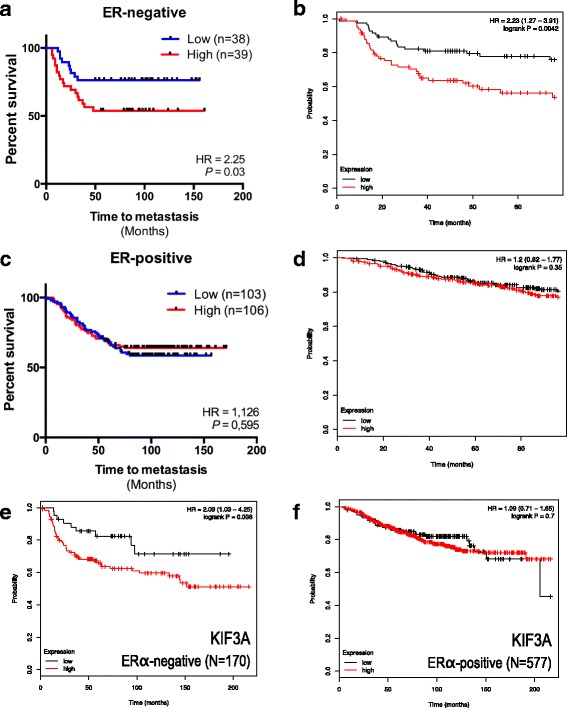
*SPEN* expression is predictive of distant metastasis-free survival in patients with ERα-negative breast cancer. **a** and **b** Kaplan-Meier graphs showing time to metastasis (**a**) and distant metastasis-free survival (**b**) in 77 and 170 patients with ERα-negative breast cancer. In both cases, the median was used as a cutoff. **c** and **d** Kaplan-Meier graphs showing time to metastasis (**c**) and distant metastasis-free survival (**d**) in 209 and 577 patients with ERα-positive breast cancer with high and low *SPEN*-expressing tumors. In both cases, the median was used as a cutoff. **e** and **f** Kaplan-Meier graphs showing distant metastasis-free survival in 170 ERα-negative (**e**) and 577 ERα-positive (**f**) patients with breast cancer with high and low *KIF3A*-expressing tumors. *ERα* Estrogen receptor alpha, *SPEN* Split ends

Cellular migration is required for cancer cells’ metastasis to distant organs [30, 31]. Because SPEN regulates cell migration in some breast cells (those HR-negative cells carrying primary cilia), the relationship between SPEN expression and time to metastasis was retrospectively investigated in cohorts of patients with early breast cancer. We found that high expression levels of *SPEN* (above the median cutoff) were predictive of early metastasis in two independent cohorts of 77 (HR 2.25, *P* = 0.03) and 170 (HR 2.23, *P* = 0.004) patients with HR-negative breast cancer (Fig. 5a and b) [32, 33]. Importantly, this association was not observed in two separate cohorts of patients with HR-positive breast cancer, possibly because HR-positive breast cancer cells typically lack primary cilia (Fig. 5c and d). Consistently, high *KIF3A* levels were also correlated with short distant metastasis-free survival in HR-negative breast cancers but not in HR-positive breast cancers, providing additional evidence that primary cilia may be linked to the metastatic process in patients with HR-negative breast cancer (Fig. 5e and f). Taken together, our findings suggest that both *SPEN* and *KIF3A* expression are associated with the development of metastasis in HR-negative breast cancers, possibly through their regulation of primary cilia levels and cellular migration.

### SPEN may regulate primary cilia formation via RFX3

**Fig. 6:**
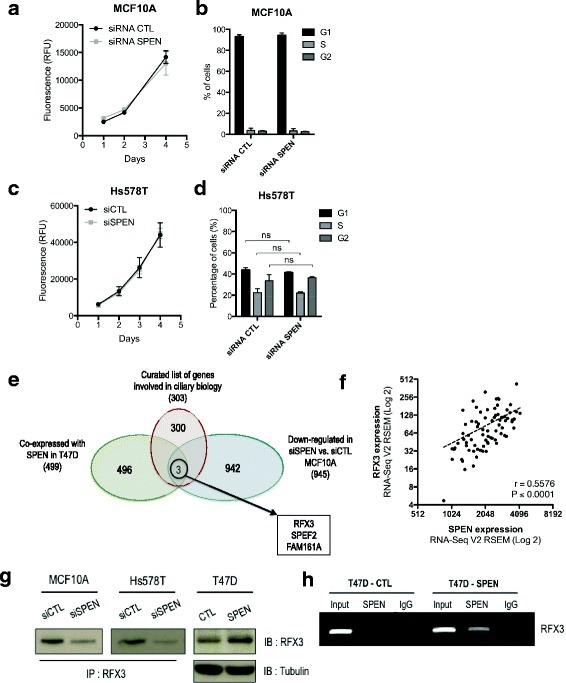
*SPEN* is coexpressed with *RFX3* in breast cancer. **a** Growth curve of MCF10A cells treated with siRNA control or siRNA for *SPEN*. Data points represent the mean fluorescence values (±SEM) of three experiments performed in quadruplicates. **b** Cell cycle analyses performed with MCF10A cells treated with siRNA control or siRNA for *SPEN*. Bar graph represents the mean percentage of cells (±SEM) in each phase of the cell cycle in three independent experiments. **c** Growth curve of Hs578T cells treated with siRNA control or siRNA for *SPEN*. Data points represent the mean fluorescence values (±SEM) of three experiments performed in quadruplicates. **d** Cell cycle analyses performed with Hs578T cells treated with siRNA control or siRNA for *SPEN*. Bar graph represents the mean percentage of cells (±SEM) in each phase of the cell cycle in three independent experiments. **e** Venn diagram showing the intersection of the list of genes coexpressed with SPEN in T47D clones, genes downregulated by *SPEN* knockdown in MCF10A cells, and a curated list of genes involved in ciliary biology. The microarray data for this study can be found on ArrayExpress under the accession numbers [E-MTAB-4974 and E-MTAB-4975]. **f** Dot plot showing that *SPEN* and *RFX3* RNA expression levels are strongly correlated in a cohort of 82 primary tumors from patients with triple-negative breast cancer. **g** Representative immunoblot (IB) showing RFX3 protein levels in T47D-CTL and T47D-SPEN as well as immunoprecipitated (IP) RFX3 protein levels in MCF10A and Hs578T cells treated with siRNA control or siRNA against *SPEN*. **h** Endpoint PCR performed on DNA immunoprecipitating with SPEN in T47D-SPEN and T47D-CTL, showing an interaction of SPEN with the *RFX3* promoter in T47D-SPEN but not in T47D-CTL cells. *** *P* ≤ 0.005; ** *P* ≤ 0.01; * *P* ≤ 0.05. *siRNA* Small interfering RNA, *SPEN* Split ends

To better define the role of SPEN in primary cilia formation and cell migration in breast cancer, we next sought to investigate mechanisms by which SPEN regulates primary ciliogenesis in breast cells. Our gene expression analysis and the previously established role of SPEN in transcriptional control led us to propose that SPEN likely modulates primary cilia incidence through the regulation of gene expression. However, on the basis of the large body of evidence indicating that primary cilia incidence is influenced by the cell cycle, we assessed whether SPEN expression affects primary cilia formation indirectly by inhibiting proliferation, which would result in a greater likelihood of primary cilia presence. We found, however, that *SPEN*-silenced MCF10A and Hs578T cells have a similar proliferative rate and distribution through the cell cycle than their respective controls (Fig. 6a–d).

To identify genes involved in ciliogenesis and potentially regulated by SPEN, we analyzed our microarray data, looking for ciliary genes upregulated by SPEN overexpression in T47D cells while consistently downregulated by SPEN knockdown in MCF10A cells (Fig. 6e). Three genes met these criteria: *SPEF2* and *FAM161A*, two structural ciliary proteins, and *RFX3*, a transcription factor and known master regulator of ciliogenesis [18, 19, 21]. To evaluate the clinical significance of these results, we assessed whether *SPEF2*, *FAM161A*, or *RFX3* RNA levels were correlated with that of SPEN in a cohort of 82 primary triple-negative tumors from The Cancer Genome Atlas database. We observed no or weak associations between *SPEN* and *SPEF2* (*R* = −0.16, *P* = 0.15) and *FAM161A* (*R* = 0.34, *P* < 0.0001) RNA levels, but we found a strong and positive correlation with *RFX3* (*R* = 0.56, *P* < 0.0001) (Fig. 6f). Of note, this positive correlation could also be observed in four cohorts of tumors from various origins, including colorectal, brain, renal, and pancreatic cancer (Additional file 4: Figure S3a–d) [34, 35]. RFX3 protein levels were validated in T47D and MCF10A cells and matched our microarray analyses (Fig. 6g) showing that SPEN reexpression resulted in higher *RFX3* RNA and protein levels and *SPEN* silencing was associated with lower *RFX3* RNA and protein levels. We also found that *SPEN* knockdown in Hs578T cells decreased RFX3 levels, suggesting that SPEN may positively regulate *RFX3* expression in different breast cancer cells (Fig. 6g). Notably, we found that SPEN is associated with the promoter region of the *RFX3* gene in T47D-SPEN but not in T47D-CTL (Fig. 6h). Altogether, these findings establish *RFX3* as a candidate transcriptional target gene of SPEN and highlight research avenues that should be explored in forthcoming studies to provide further mechanistic insights into the role of SPEN in primary ciliogenesis.

## Discussion

Primary cilia have long been considered as organelles of little to no functional and biological relevance. However, it is now clear that primary cilia play important roles during development, with ciliary dysfunction being associated with a variety of diseases, including polydactyly, brain malformations, situs inversus (abnormal left-right patterning), and polycystic kidney disease. More recently, primary cilia were also shown to be required for appropriate branching morphogenesis during the maturation of the breast, a bilayered ductal system composed of apically oriented luminal epithelial cells encircled by contractile and basement membrane-associated basal cells [36, 37]. Interestingly, this study uncovered that primary cilia are found on both luminal and basal cells during branching morphogenesis but disappear from luminal cells after completion of breast development. Consistently, analysis of reduction mammoplasties from 12 women revealed that primary cilia are highly prevalent in basal (median 23.6%) but not luminal (median 1.1%) breast epithelial cells [10]. Altogether, the data reported in these studies indicate that primary cilia may be more functionally and biologically important in basal than in luminal breast cells.

In the present study, we have established a novel role for SPEN in the regulation of primary cilia formation in breast epithelial cells. Indeed, we report that SPEN silencing reduces primary cilia formation in MCF10A and Hs578T cells, two cell types known to harbor high levels of primary cilia. Although luminal breast cancer cells are typically nonciliated, it should not be excluded that SPEN may also regulate primary cilia formation in these cells, as observed in our T47D model. Indeed, SPEN reexpression in T47D cells was able to restore the primary cilia to a level that is within the range previously reported for normal luminal breast epithelial cells (median 1.1%) and breast cancer cells (0.3–3.3%) [9]. It is possible that characteristics intrinsic to luminal cells, including their unique gene expression profile and anatomical location, are responsible for their low abundance of primary cilia. This may also provide an explanation for the very low degree of restored primary cilia expression in T47D-SPEN cells to a level that is within the intrinsic capacity range of luminal breast cell populations.

Although identified more than 100 years ago, primary cilia are cellular structures whose functions in normal and cancer cells remain elusive. Indeed, it has been proposed that primary cilia have tumor-suppressive functions because they decrease in incidence in cancer compared with normal tissues. Yet, a large body of evidence also indicates that primary cilia may have tumor-promoting effects, owing to their ability to sense and integrate signals from oncogenic pathways, including the Hedgehog, Wnt, and PDGFR transduction pathways. As suggested by others, primary cilia may have dual roles in cancer development, functioning as tumor suppressors early in cancer formation but conferring aggressiveness at later stages of the disease through the regulation of oncogenic signaling pathways [38–40].

In addition to its regulation of primary cilia formation, we also report that SPEN coordinates migration in breast epithelial cells, but only in those that are ciliated. Indeed, we found a correlation between SPEN-mediated effects on migration and primary cilia abundance, suggesting that SPEN may control migration through primary cilia-dependent mechanisms. To evaluate the clinical significance of these results, we assessed whether SPEN expression levels are correlated with metastasis in breast cancer and found that high *SPEN* RNA expression levels are predictive of early metastasis in two independent cohorts of ERα-negative but not ERα-positive breast cancers. These clinical findings are consistent with the very low abundance of primary cilia in luminal and ERα-positive breast cancer cells in vitro. However, it is important to note that cilia expression is very low in clinical breast cancer samples and not significantly different between ERα-positive and ERα-negative breast tumors. This may be explained by the 3D nature of primary tissues and the altered structural architecture of cancer compared with normal tissues, which may render the assessment of primary cilia levels in clinical samples more difficult and imprecise than for cultured cells. Preclinical studies with mouse models may provide further clues to better understand the role of primary cilia, the incidence of which is influenced by SPEN levels, in the metastatic process in breast cancer.

Metastasis is a very complex multistep process that imposes many more barriers and challenges to cancer cells than a requirement for migration. Yet, several lines of evidence are compatible with a role for SPEN through its regulation of primary cilia abundance and migration in one or several steps in the metastatic process. For instance, Emoto et al. [12] recently demonstrated that patients with primary cilia-positive pancreatic cancers have a higher probability of developing lymph node metastasis than those whose tumors are nonciliated. In their report, they further showed that primary cilia expression constitutes an independent poor prognostic factor of overall patient survival in pancreatic cancer (HR 3.47, *P* = 0.01) [12]. Such findings are compatible with a role for primary cilia in cancer cells dissemination to distant organ sites and may explain why *SPEN* expression is linked to metastasis in patients with ERα-negative breast cancers.

Although this study was focused on addressing the role of SPEN in primary cilia formation in breast cancer, it highlights the functions of SPEN in primary ciliogenesis that may also apply to other cell types, as seen in fibroblasts, and possibly many more cancer types. With hormone-dependent and hormone-independent functions, SPEN is a complex protein whose roles with regard to the establishment of primary cilia should be further investigated, especially in skin, pancreatic, and lung cancers, which are aggressive and highly metastatic types of cancer. In general, the results presented in this study reveal new functions for SPEN in the regulation of primary cilia formation and migration in breast cells. In addition, they shed light on research avenues that deserve further consideration in the field of cancer metastasis, which is still responsible for more than 90% of cancer-related deaths.

## Conclusions

We have demonstrated that SPEN regulates primary cilia formation and cellular migration in ciliated ERα-negative breast cancer cells and that *SPEN* expression levels are associated with early metastasis in patients with HR-negative breast cancers. These hormone-independent functions of SPEN suggest that SPEN expression plays a significant role in the biology of ERα-negative breast tumors.

## Additional files

Additional file 1: Table S1. List of ciliary genes coexpressed with SPEN in T47D and MCF10A cells. (PDF 31 kb)

Additional file 2: Figure S1. MCF10A cells grown with or without cholera toxin display important phenotypic and functional differences. (**a** and **b**) Representative pictures of MCF10A cells grown in the absence (**a**) or presence (**b**) of cholera toxin. (**c** and **d**) Representative images of primary cilia in MCF10A cells grown in the absence (**c**) or presence (**d**) of cholera toxin. *Arrows* point to primary cilia (scale bar = 5 μm). (**e**) Cell cycle analyses performed with MCF10A cells grown in the absence and presence of cholera toxin. Bar graph represents the mean percentage of cells (±SEM) in each phase of the cell cycle in three independent experiments. (**f**) Growth curve of MCF10A cells grown in the presence or absence of cholera toxin. Data points represent the mean fluorescence values (±SEM) of three experiments performed in quadruplicates. (PDF 9130 kb)

Additional file 3: Figure S2. SPEF2 silencing in T47D-SPEN cells inhibits cellular migration. (**a**) Western blot analysis of SPEF2 protein levels in T47D-SPEN cells treated with a CTL siRNA (siCTL) or two different SPEF2 siRNAs (siSPEF2#1 and #2) and T47D-CTL cells treated with a CTL siRNA (siCTL; two independent lysates). (**b**) Effect of SPEF2 knockdown on the migration of T47D SPEN cells was evaluated by performing Transwell migration assays. Each error bar represents the mean and SEM of three independent experiments performed in duplicate. *p < 0.05, **p < 0.01. (**c**) Representative images of migrated cells fixed and stained with crystal violet after 72 h. (PDF 456 kb)

Additional file 4: Figure S3. RFX3 levels correlate with SPEN levels across many cancer types. (**a–d**) Dot plots showing that *SPEN* and *RFX3* RNA expression levels are strongly correlated in cohorts of colon (**a**), brain (**b**), pancreatic (**c**), and renal (**d**) cancers. (PDF 3707 kb)

## Abbreviations

ATCC: American Type Culture Collection
cRNA: Complementary RNA
CT: Cholera toxin
DAPI: 4′,6-Diaminodino-2-phenylindole
EDTA: Ethylenediaminetetraacetic acid
ERα: Estrogen receptor alpha
GO: Gene Ontology
HR: Hormone receptor
IgG: Immunoglobulin G
PDGFR: Platelet-derived growth factor receptor
RNAi: RNA interference
SHH: Sonic hedgehog
siRNA: Small interfering RNA
SPEF2: Sperm flagellar 2
SPEN: Split ends
SPOC: Spen paralogue and orthologue C-terminal domain
