# Supplementary material for: SPEN, a new player in primary cilia formation and cell migration in breast cancer
Source: Breast Cancer Res. 2017 Sep 6;19:104. doi: 10.1186/s13058-017-0897-3 (PMC5588740; doi:10.1186/s13058-017-0897-3)
Supplement: Supplementary file 1 — List of ciliary genes coexpressed with SPEN in T47D and MCF10A cells. (PDF 31 kb) [file 13058_2017_897_MOESM1_ESM.pdf]

T47D
AK7
AK8
ARL6
B9D2
BBS5
CC2D2A
CCDC103
CCDC114
CCDC39
CCDC40
DNAAF1
DNAH2
DNAH5
DNAH6
DNAI1
FAM161A
FOXJ1
HYDIN
LRRC6
MAK
NME5
NPHP1
RFX3
ROPN1L
RPGR
RSPH1
RSPH4A
RSPH9
SPEF2
TEKT2
TMEM231
TMEM67
TTC26
TTLL9
WDR35
WDR78

MCF10A
CCDC41
CEP97
DISC1
DPCD
FAM161A
KIF24
MNS1
NEK2
NUP35
ORC1
PLK1
POC1A
PTCH1
RAB17
RAB3IP
RFX3
SASS6
SPA17
SPEF2
STIL
TCTN2
TRAPPC3
TTK
USH1G
WDR60
