# Supplementary figures and images for: SPEN, a new player in primary cilia formation and cell migration in breast cancer
Source: Breast Cancer Res. 2017 Sep 6;19:104. doi: 10.1186/s13058-017-0897-3 (PMC5588740; doi:10.1186/s13058-017-0897-3)

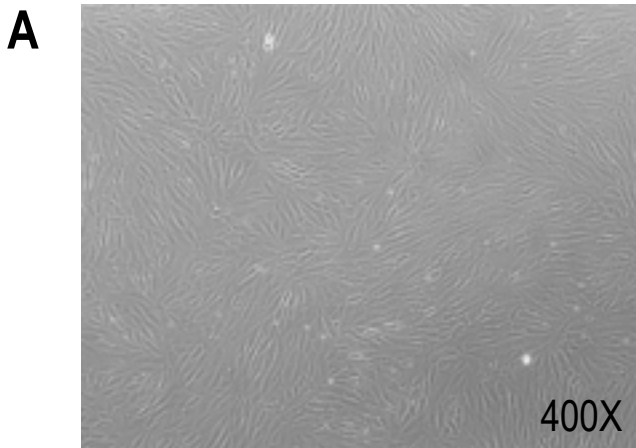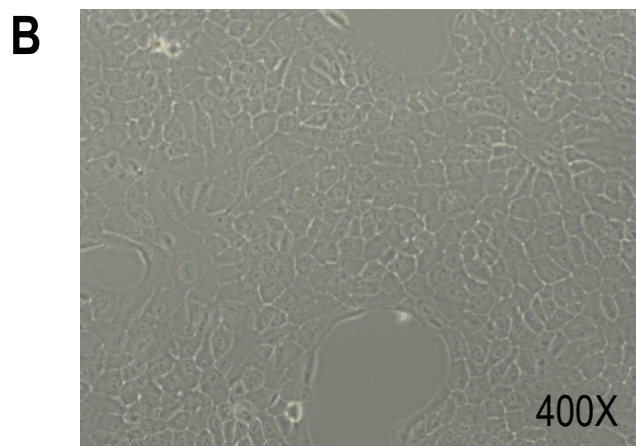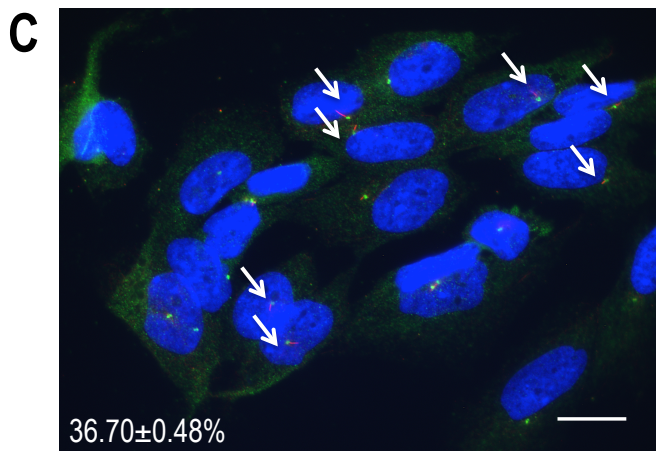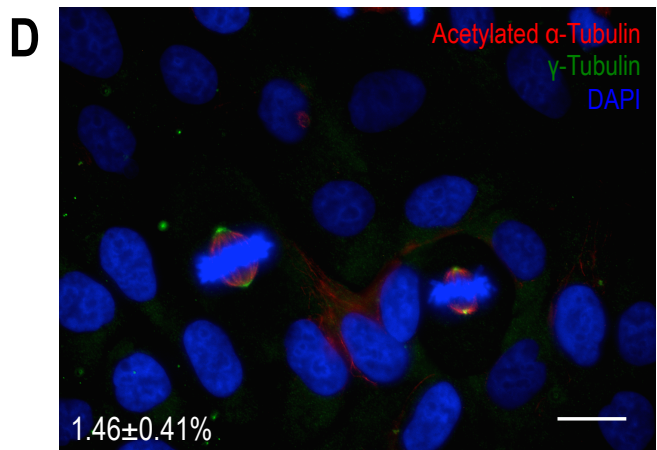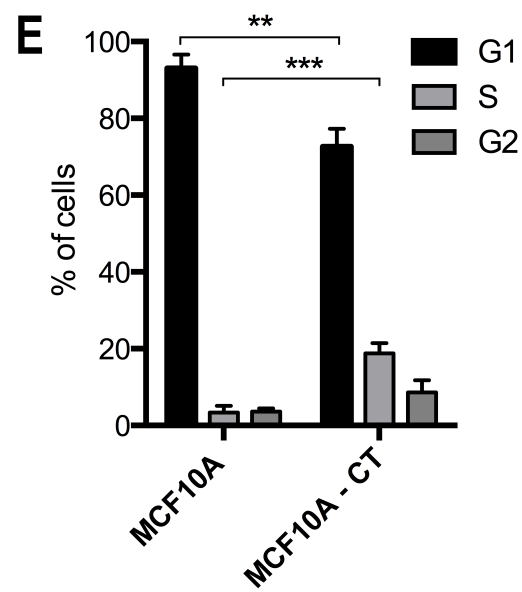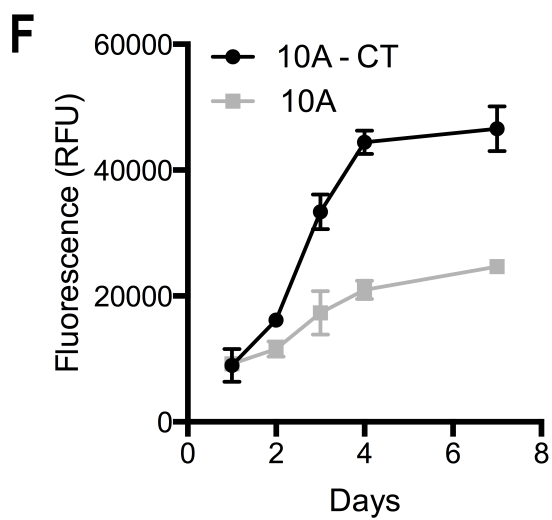

Supplement: Supplementary file 2 — MCF10A cells grown with or without cholera toxin display important phenotypic and functional differences. (a and b) Representative pictures of MCF10A cells grown in the absence (a) or presence (b) of cholera toxin. (c and d) Representative images of primary cilia in MCF10A cells grown in the absence (c) or presence (d) of cholera toxin. Arrows point to primary cilia (scale bar = 5 μm). (e) Cell cycle analyses performed with MCF10A cells grown in the absence and presence of cholera toxin. Bar graph represents the mean percentage of cells (±SEM) in each phase of the cell cycle in three independent experiments. (f) Growth curve of MCF10A cells grown in the presence or absence of cholera toxin. Data points represent the mean fluorescence values (±SEM) of three experiments performed in quadruplicates. (PDF 9130 kb) [file 13058_2017_897_MOESM2_ESM.pdf]

# Figure S2

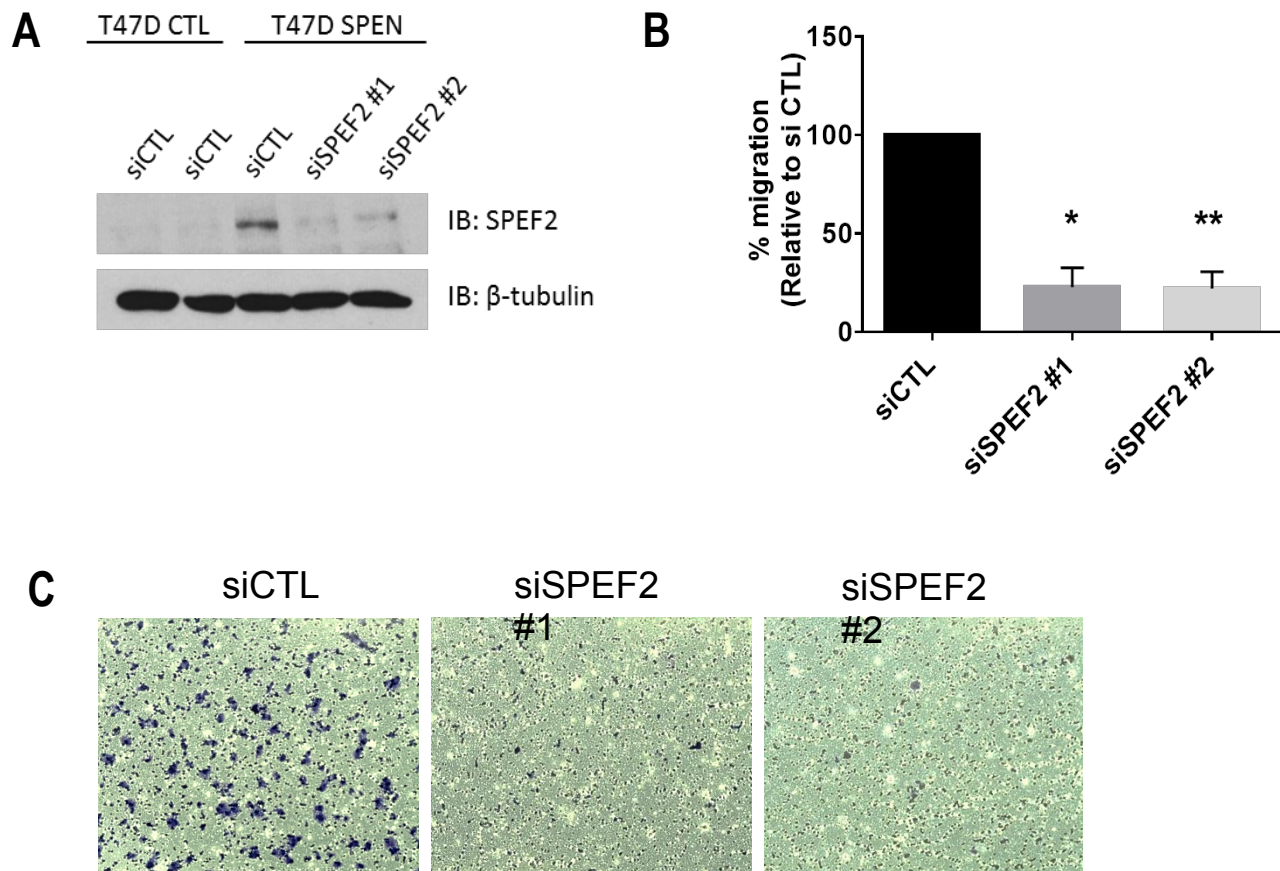

Supplement: Supplementary file 3 — SPEF2 silencing in T47D-SPEN cells inhibits cellular migration. (a) Western blot analysis of SPEF2 protein levels in T47D-SPEN cells treated with a CTL siRNA (siCTL) or two different SPEF2 siRNAs (siSPEF2#1 and #2) and T47D-CTL cells treated with a CTL siRNA (siCTL; two independent lysates). (b) Effect of SPEF2 knockdown on the migration of T47D SPEN cells was evaluated by performing Transwell migration assays. Each error bar represents the mean and SEM of three independent experiments performed in duplicate. *p < 0.05, **p < 0.01. (c) Representative images of migrated cells fixed and stained with crystal violet after 72 h. (PDF 456 kb) [file 13058_2017_897_MOESM3_ESM.pdf]

**Figure S3**

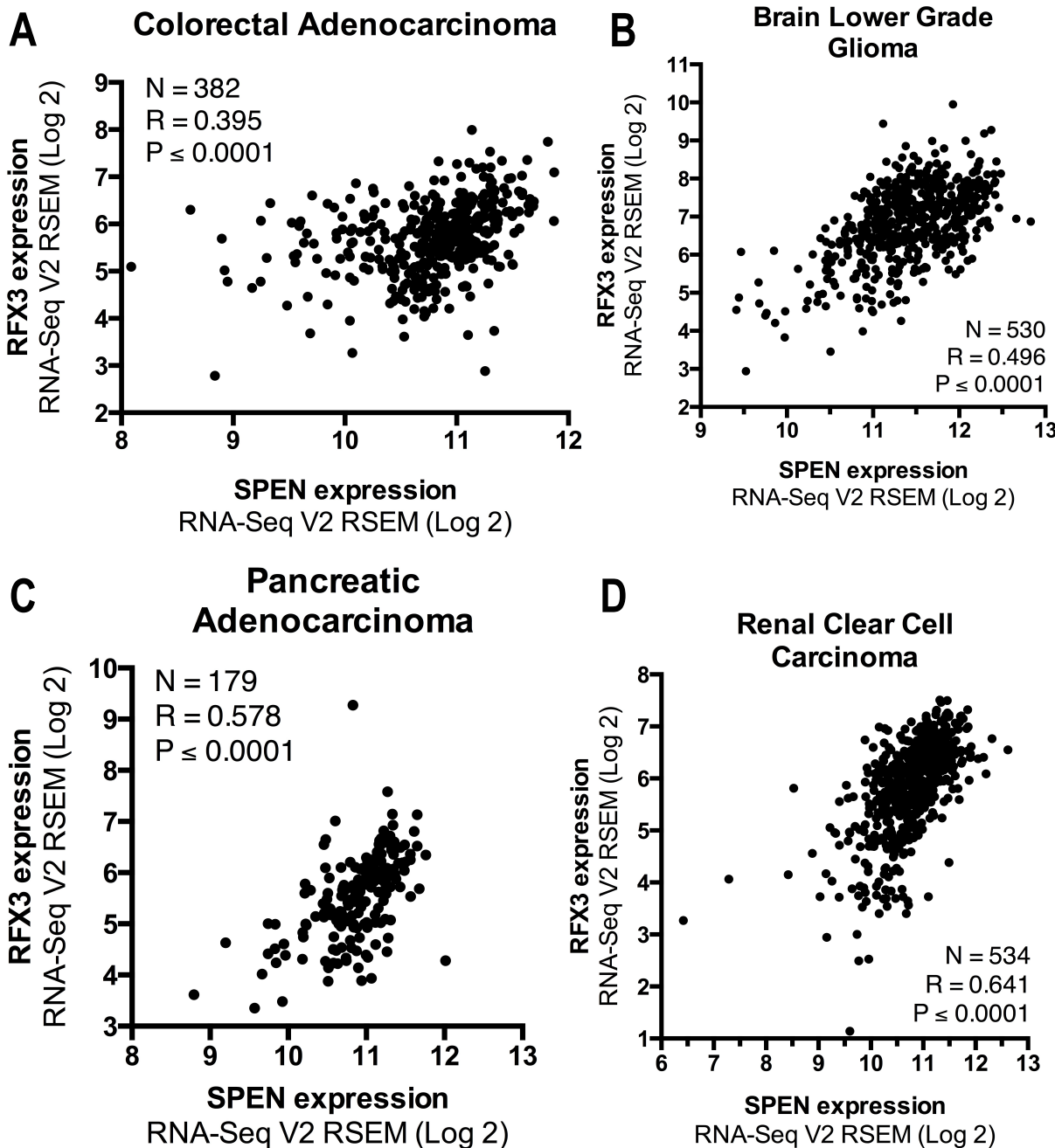

Supplement: Supplementary file 4 — RFX3 levels correlate with SPEN levels across many cancer types. (a–d) Dot plots showing that SPEN and RFX3 RNA expression levels are strongly correlated in cohorts of colon (a), brain (b), pancreatic (c), and renal (d) cancers. (PDF 3707 kb) [file 13058_2017_897_MOESM4_ESM.pdf]
